# Survey of Anaphylaxis during Rasburicase Re-Administration in Patients with Hematological Malignancies Using a Japanese Claims Database

**DOI:** 10.3390/curroncol29120772

**Published:** 2022-12-14

**Authors:** Shunsuke Kobayashi, Takeo Yasu, Manabu Akazawa

**Affiliations:** 1 Department of Public Health and Epidemiology, Meiji Pharmaceutical University, Tokyo 204-8588, Japan; 2 Department of Pharmacy, Nissan Tamagawa Hospital, Tokyo 158-0095, Japan; 3 Pharmaceutical Education and Research Center, Department of Medicinal Therapy Research, Meiji Pharmaceutical University, Tokyo 204-8588, Japan

**Keywords:** rasburicase, anaphylaxis, re-administration, hematological malignancies, claims data

## Abstract

Management of tumor lysis syndrome (TLS) associated with cancer chemotherapy for malignant tumors is important because of its potentially fatal course. The use of rasburicase, a recombinant urate oxidase, is recommended for TLS; however, because rasburicase is an enzymatic drug, one should be cautious of anaphylaxis during administration. Using claims data in Japan, we investigated the rate of rasburicase re-administration and the occurrence of anaphylaxis during re-administration in patients with hematopoietic malignancies in a multicenter setting. Re-administration of rasburicase was defined as administration after an interval of 21 days from the first dose. Of 373 patients, 18 were re-administered rasburicase (re-administration rate: 4.8%). No patient developed anaphylaxis. The median number of days from the first to the last dose of rasburicase was 256.5 days (interquartile range: 138.8–455.8 days). The median daily dose was 7.5 mg (4.5–11.3 mg), and the median total dose was 33.8 mg (19.1–64.1 mg). This claims database analysis revealed that the re-administration rate of rasburicase was low in Japanese patients with hematopoietic malignancies, suggesting that rasburicase was being used appropriately, and that associated anaphylaxis was not observed.

## 1. Introduction

Tumor lysis syndrome (TLS) is a metabolic disorder caused by rapid destruction of tumor cells and is induced by cancer chemotherapy for malignant tumors. It can lead to a fatal outcome; hence, its management is important [1]. TLS often occurs in patients with hematopoietic malignancies (such as malignant lymphomas and acute leukemia) and results from rapid decay of tumor cells that are highly sensitive to chemotherapy. Uric acid levels increase over a short duration in TLS [2]. In addition to the uric acid production inhibitors allopurinol and febuxostat, the use of the recombinant urate oxidase rasburicase is recommended for the management of TLS [1,2]. In the majority of adult patients with hematopoietic malignancies that are associated with a high risk of TLS but who do not meet the diagnostic criteria for laboratory or clinical TLS, TLS can be prevented by administering a single dose of rasburicase with subsequent close monitoring [2].

Conversely, rasburicase is an enzymatic drug, and its administration produces antibodies; thus, it is necessary to monitor for and manage rasburicase-induced anaphylaxis appropriately. Japanese clinical trials have confirmed antibody production in 10% of patients treated with rasburicase [3]. Allen et al. conducted a single-center retrospective analysis and reported anaphylaxis in 6.2% of the patients in whom rasburicase was re-administered [4]. However, only a few studies have reported the occurrence of anaphylaxis after re-administration of rasburicase along with studies on multicenter and claims data [4,5]. In addition, according to the US Food and Drug Administration, the clearance rate of rasburicase in the Japanese population is approximately 40% lower than that in Caucasians; hence, it is necessary to verify its safety among the Japanese population [6].

Therefore, in this study, we survey the rate of re-administration of rasburicase and the occurrence of anaphylaxis during re-administration in patients with hematopoietic malignancies at multiple centers in Japan using claims data.

## 2. Materials and Methods

### 2.1. Study Design and Patients

In this retrospective cohort study, we used the JMDC database [7]. This is a nationwide epidemiological claims database in Japan that accumulates claims data (hospitalization, outpatient, and dispensing) collected from multiple health insurance associations. All patient data are encrypted before addition to the database. Data in the JMDC database include disease codes (International Classification of Diseases-10 (ICD-10)), basic patient information, prescription history, and details of medical care. However, laboratory data are not included.

Between January 2005 and September 2019, 21,489 patients with hematopoietic malignancies (ICD-10 codes C81–C96 and D46) had their data recorded in the database. Among these patients, those aged 15 years or older who underwent rasburicase re-administration were included in this study.

According to the ethical guidelines for epidemiological research in Japan, if informed consent cannot be obtained due to the method or content of the epidemiological research, it must be approved by the ethics committee and permitted by the director of the research institution, and thus, its requirement can be waived. In addition, for observational research that only uses previously acquired data, it is not always necessary to obtain informed consent from the research participant; however, information regarding how the research was conducted must be disclosed. Therefore, with the approval of our Institutional Ethics Board, we provided the participants the option to opt out of the study on our laboratory homepage. Since this study is a database study, we did not obtain patient informed consent.

### 2.2. Data Collection and Variable Definition

Patient data were collected following the administration of the first dose of rasburicase. In accordance with the definition provided by Allen et al., re-administration of rasburicase was defined as the administration of rasburicase after an interval of 21 days from its initial administration [5]. The monitoring period for anaphylaxis extended from the day of rasburicase re-administration to 3 days after exposure when biphasic anaphylaxis was reported [8]. Anaphylaxis was defined as being assigned the ICD-10 codes for anaphylaxis, either T782 (unspecified anaphylactic shock) or T886 (anaphylactic shock as a side effect of an appropriate drug or a drug properly administered), and being prescribed adrenaline injections during this period [9,10].

The Charlson Comorbidity Index (CCI) score was based on the ICD-10 code reported by Quan et al. [11,12]. CCI scores were extracted for the following comorbidities: myocardial infarction, congestive heart failure, peripheral arterial disease, cerebrovascular disease, dementia, connective tissue disease, peptic ulcer disease, mild liver disease, moderate or severe liver disease, diabetes with or without chronic complications, chronic pulmonary disease, hemiplegia or paraplegia, renal disease, malignancies, metastatic solid tumor, and acquired immunodeficiency syndrome/human immunodeficiency virus infection.

Patient characteristics were baselined at the first administration of rasburicase. Data extracted included the age, sex, all hematopoietic malignancies, CCI scores and associated comorbidities, antineoplastic drugs, concomitant use of allopurinol and febuxostat (uric acid production inhibitors), doses, number of courses, and days of rasburicase administration, and number of days between the first and last rasburicase administration.

The rasburicase re-administration rate and incidence of anaphylaxis in the re-administered patients were calculated. Values are presented as the number of cases (%) or median (interquartile range (IQR)).

## 3. Results

**Figure 1:**
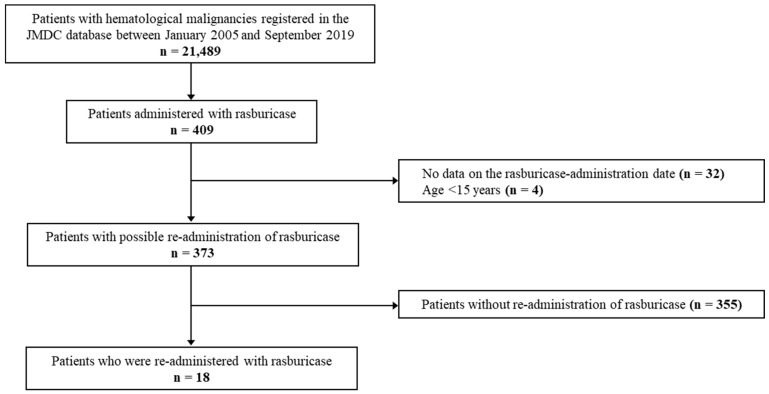
Flow diagram of patient selection.

Rasburicase was prescribed to 409 patients, of whom 36 were excluded from the analysis. Rasburicase was then re-administered to 18 of the remaining 373 patients (Figure 1).

The rasburicase re-administration rate was 4.8%. No patient was assigned an ICD-10 code for anaphylactic shock. One patient was prescribed adrenaline injections (given the ICD-10 code for septic shock) and also received antibiotics. Therefore, no patient developed anaphylaxis.

The median age at baseline was 56 years (36–59 years). The study population comprised 12 men (66.7%). Hematopoietic malignancies were mainly acute lymphocytic leukemia in five cases (27.8%), acute myeloid leukemia in three cases (16.7%), and Mantle cell lymphoma and non-Hodgkin’s lymphoma in two cases (11.1%). The median CCI score was 5.5 (3.8–9.3), and 12 patients (66.7%) had a CCI score ≥5; thus, the number of high-risk patients was high. Comorbidities associated with the CCI score were mainly mild liver disease in 13 cases (66.7%); peptic ulcer disease in 9 cases (50%); congestive heart failure, diabetes without chronic complications, and chronic pulmonary disease in 8 cases (44.4%); and metastatic solid tumors in 5 cases (27.8%). The following antineoplastic drugs were administered: cytarabine (n = 9; 50.0%); cyclophosphamide, vincristine, and doxorubicin (n = 6 each; 33.3%); and rituximab (n = 4; 22.2%). Allopurinol and febuxostat were co-administered in four (22.2%) and two (11.1%) patients, respectively.

The median number of courses of rasburicase was 2 (2–4). Furthermore, the median total days of rasburicase administration was 5.5 days (3.8–9.8 days). The median number of days between administration of the first and last doses was 256.5 days (138.8–455.8 days). The median dose administered per day was 7.5 mg (4.5–11.3 mg/day), while the median total dose was 33.8 mg (19.1–64.1 mg). The patient characteristics are summarized in Table 1.

## 4. Discussion

To the best of our knowledge, this is the first survey performed to verify the re-administration rate of rasburicase and the associated incidence of anaphylaxis using claims data. This survey was conducted at 23 facilities, a number that is approximately three times larger than the number of facilities involved in clinical trials in Japan [3].

The JMDC database comprises real-world medical practice data, including information on medical fee statements. All medical fee claims at insurance medical institutions are collected by the insurer; thus, even if a patient receives treatment at multiple medical institutions, all medical treatment details can be obtained using the database [7]. Rasburicase is an expensive drug, and its usage is covered by health insurance. Because the JMDC database can track the prescription history of rasburicase, it is considered a suitable database for verifying the safety of re-administration. In addition, rasburicase is administered infrequently; the number of days of administration is limited to five (seven in Japan), and re-administration is not recommended [6]. Therefore, collecting re-administration cases for analyses is a difficult task. Hence, we conducted this study using the claims database. However, since the JMDC database is a claims database, it does not store detailed medical information.

TLS is caused by the destruction of tumor cells; nucleic acids are metabolized to xanthine and subsequently uric acid, resulting in hyperuricemia. In addition, hyperphosphatemia and hyperkalemia occur concurrently. When blood pressure decreases and inflammation occurs due to cytokines, acute renal failure develops. Subsequently, the amounts of uric acid, potassium, and phosphorous exceed the urinary excretion capacity, and large amounts of these materials suddenly enter the blood and are released into the body. This can lead to various TLS pathologies, including renal failure, arrhythmia, seizures, and even death [1]. Rasburicase is a recombinant urate oxidase that metabolizes uric acid to allantoin. This metabolism is rapid; because the urinary solubility of allantoin is extremely high as compared with that of uric acid, the blood uric acid concentration is lowered rapidly. Therefore, rasburicase is recommended in patients at high risk of TLS [2]. Conventional uric acid production inhibitors, such as allopurinol, are incapable of lowering pre-existing elevated uric acid levels in the presence of acute kidney injury, thereby making rasburicase an important drug in such conditions [1]. However, although urate oxidase is an endogenous enzyme commonly found in most mammalian species, it is absent in humans due to the acquisition of nonsense mutations in the coding region during human evolution. Therefore, administration of rasburicase may lead to the production of antibodies, which may induce anaphylaxis [13]. However, the mechanism of anaphylaxis development during re-administration of rasburicase has not yet been elucidated.

Allen et al. reported that the incidence of anaphylaxis after re-administration of rasburicase was 6.2%; however, anaphylaxis did not occur in any patient in our study [4]. In Japan, the re-administration rate of rasburicase is as low as 4.8%, which indicates that it is being administered appropriately. Allen et al. reported that multiple myeloma and acute leukemia accounted for 47.4% and 22.7% of the cases of hematopoietic malignancies in their study, respectively, and that five of the six patients who developed anaphylaxis had multiple myeloma. Conversely, no patients with multiple myelomas were included in our study, whereas 44.4% of the patients had acute leukemia. The recommended dosage of rasburicase on the label is 0.20 mg/kg [6]. The daily dose in the present study was 7.5 mg (4.5–11.3 mg); conversely, Allen et al. reported daily doses of 3.5 mg (3–6 mg) and 3.07 mg (3–4 mg) for the anaphylaxis and non-anaphylaxis groups, respectively (which were more than twice the dose in our study) [4]. The JMDC database does not contain information on patients’ weights; however, according to Japanese health statistics, the average weights of men and women aged 50–59 years in 2018 were 70.4 kg and 55.2 kg, respectively [14]. Allen et al. reported mean body weights of 86.85 kg (55.6–124.6 kg) and 85.7 kg (47.3–164 kg) in the anaphylaxis and non-anaphylaxis groups, respectively. Therefore, we believe that the dosage per body weight was higher in our study than in the study by Allen et al. The total dose was 33.8 mg (19.1–64.1 mg) in this study, while Allen et al. reported a total dose of 6–18 mg in the anaphylaxis group in their study; therefore, the dose was higher in our study [4]. In addition, it has been reported that rasburicase clearance in the Japanese is approximately 40% lower than that in Caucasians [6]. Based on these findings, the amount of exposure to rasburicase in this study is considered to be much higher than that reported by Allen et al.; however, anaphylaxis did not occur. Thus, we speculate that the incidence of anaphylaxis after re-administration of rasburicase may be lower in Japanese individuals than in Caucasians [4].

In this study, 4 out of 18 patients received rasburicase at multiple centers: 3 patients received rasburicase at two centers and 1 received it at three centers. It is unclear whether the treatment histories of these patients were shared between the centers. In order to prevent anaphylaxis due to rasburicase, it is necessary to understand the administration history. Therefore, it is important to interview the patients, prepare medical information documents, and direct medical personnel to provide support to ensure that the patients themselves can maintain their treatment history.

In clinical practice, it is assumed that rasburicase re-administration may be required during repeated courses of cancer chemotherapy in patients at a high risk of TLS. In a Japanese clinical trial, an antibody measurement test revealed no antibody production from the start of rasburicase administration (0.20 mg/kg) to day 8 after administration. However, antibodies were confirmed in 12% and 8% of the patients on day 29 and at 6 months (±2 months) after administration, respectively [3]. The risk of immunogenicity is known to increase with higher doses and longer treatment periods; thus, strict monitoring for anaphylaxis is still necessary while re-administering rasburicase to Japanese patients [15]. Consideration of premedication with antihistamines and corticosteroids, preparation of adrenaline injections, establishment of a system that can quickly undertake respiratory and circulatory management, and continued strict monitoring can prevent the occurrence of anaphylaxis. Preparation in advance is important.

This study has some limitations. First, the number of patients was small. However, it is necessary to accumulate such evidence one by-one. Second, rigorous verification by weight-equivalent dosage was not possible due to the lack of information on the patients’ body weight. Third, the JMDC database is mainly limited to data on employees of large companies and their families under the age of 75 years; hence, the development of anaphylaxis in the elderly, employees of small and medium enterprises, and self-employed people and their families have not been evaluated.

## 5. Conclusions

Claims database analysis revealed that the re-administration rate of rasburicase was low in patients with hematopoietic malignancies in Japan; anaphylaxis was not observed following rasburicase re-administration.

## Figures and Tables

**Table 1:** Baseline patient characteristics.

	n = 18
Age (years), median (IQR)	56 (36–59)
Sex, male n (%)	12 (66.7)
Hematological malignancies, n (%)	
Acute lymphoblastic leukemia	5 (27.8)
Acute myeloid leukemia	3 (16.7)
Mantle cell lymphoma	2 (11.1)
Non-Hodgkin lymphoma	2 (11.1)
Mixed leukemia	1 (5.6)
Myelodysplastic syndrome	1 (5.6)
Plasmablastic lymphoma	1 (5.6)
Burkitt lymphoma	1 (5.6)
Plasma cell myeloma	1 (5.6)
Malignant lymphoma	1 (5.6)
Charlson Comorbidity Index score, median (IQR)	5.5 (3.8–9.3)
Charlson Comorbidity Index score of ≥5, n (%)	12 (66.7)
Charlson Comorbidity Index Comorbidities, n (%)	
Malignancies	18 (100)
Mild liver disease	13 (72.2)
Peptic ulcer disease	9 (50.0)
Congestive heart failure	8 (44.4)
Diabetes without chronic complications	8 (44.4)
Chronic pulmonary disease	8 (44.4)
Metastatic solid tumor	5 (27.8)
Others	6 (33.3)
Chemotherapy regimens, n (%)	
Cytarabine	9 (50.0)
Cyclophosphamide	6 (33.3)
Vincristine	6 (33.3)
Doxorubicin	6 (33.3)
Etoposide	5 (27.8)
Rituximab	4 (22.2)
Methotrexate	4 (22.2)
Mitoxantrone	4 (22.2)
Others	11 (61.1)
Concomitant use of anti-hyperuricemia drugs, n (%)	
Allopurinol	4 (22.2)
Febuxostat	2 (11.1)
Rasburicase administrations, median (IQR)	
Number of courses	2 (2–4)
Days of administration for each course	2 (1.5–4.5)
Total days of administration	5.5 (3.8–9.8)
Days from first administration to last administration	256.5 (138.8–455.8)
Dose (mg/day)	7.5 (4.5–11.3)
Total dose (mg)	33.8 (19.1–64.1)
IQR: interquartile range	

## Data Availability

No new data were created or analyzed in this study. Data sharing is not applicable to this article.

## References

[1] Howard S.C., Jones D.P., Pui C.H. (2011). The Tumor Lysis Syndrome. N. Engl. J. Med..

[2] Jones G.L., Will A., Jackson G.H., Webb N.J., Rule S., British Committee for Standards in Haematology (2015). Guidelines for the Management of Tumour Lysis Syndrome in Adults and Children with Haematological Malignancies on Behalf of the British Committee for Standards in Haematology. Br. J. Haematol..

[3] Ishizawa K., Ogura M., Hamaguchi M., Hotta T., Ohnishi K., Sasaki T., Sakamaki H., Yokoyama H., Harigae H., Morishima Y. (2009). Safety and Efficacy of Rasburicase (SR29142) in a Japanese Phase II Study. Cancer Sci..

[4] Allen K.C., Champlain A.H., Cotliar J.A., Belknap S.M., West D.P., Mehta J., Trifilio S.M. (2015). Risk of Anaphylaxis with Repeated Courses of Rasburicase: A Research on Adverse Drug Events and Reports (RADAR) Project. Drug Saf..

[5] Alaya S., Mofredj A., Darmon N., Tassaioust K., Mrabet A. (2018). Anaphylactic Shock Following Repeated Rasburicase Treatment. Ann. Pharmacother..

[6] Rasburicase Manufacturer’s Information December 2019. https://www.accessdata.fda.gov/drugsatfda_docs/label/2019/103946s5103lbl.pdf.

[7] Japan Medical Data Center. https://www.jmdc.co.jp/en/.

[8] Tole J.W., Lieberman P. (2007). Biphasic Anaphylaxis: Review of Incidence, Clinical Predictors, and Observation Recommendations. Immunol. Allergy Clin. N. Am..

[9] Tadokoro F., Morita K., Michihata N., Fushimi K., Yasunaga H. (2018). Association between Sugammadex and Anaphylaxis in Pediatric Patients: A Nested Case-Control Study Using a National Inpatient Database. Paediatr. Anaesth..

[10] Simons F.E., Ardusso L.R., Bilò M.B., El-Gamal Y.M., Ledford D.K., Ring J., Sanchez-Borges M., Senna G.E., Sheikh A., Thong B.Y. (2011). World Allergy Organization Guidelines for the Assessment and Management of Anaphylaxis. World Allergy Organ. J..

[11] Charlson M.E., Charlson R.E., Peterson J.C., Marinopoulos S.S., Briggs W.M., Hollenberg J.P. (2008). The Charlson Comorbidity Index is Adapted to Predict Costs of Chronic Disease in Primary Care Patients. J. Clin. Epidemiol..

[12] Quan H., Sundararajan V., Halfon P., Fong A., Burnand B., Luthi J.C., Saunders L.D., Beck C.A., Feasby T.E., Ghali W.A. (2005). Coding Algorithms for Defining Comorbidities in ICD-9-CM and ICD-10 Administrative Data. Med. Care.

[13] Yeldandi A.V., Yeldandi V., Kumar S., Murthy C.V., Wang X.D., Alvares K., Rao M.S., Reddy J.K. (1991). Molecular Evolution of the Urate Oxidase-Encoding Gene in Hominoid Primates: Nonsense Mutations. Gene.

[14] Japan’s Ministry of Health, Labor and Welfare Statistical Information, Average Height and Weight, by Gender, Year, Age. https://view.officeapps.live.com/op/view.aspx?src=https%3A%2F%2Fwww.mhlw.go.jp%2Ftoukei%2Fyouran%2Fdatar03k%2F2-06.xlsx&wdOrigin=BROWSELINK.

[15] Schellekens H. (2005). Factors Influencing the Immunogenicity of Therapeutic Proteins. Nephrol. Dial. Transplant..

